# The Dark Side of EGFP: Defective Polyubiquitination

**DOI:** 10.1371/journal.pone.0000054

**Published:** 2006-12-20

**Authors:** Mathijs Baens, Heidi Noels, Vicky Broeckx, Sofie Hagens, Sabine Fevery, An D. Billiau, Hugo Vankelecom, Peter Marynen

**Affiliations:** 1 Applied Human Genomics, Center for Human Genetics, Molecular Genetics ‐ Flanders Interuniversity Institute for Biotechnology (VIB), University of Leuven Leuven, Belgium; 2 Laboratory of Experimental Transplantation, University of Leuven Leuven, Belgium; 3 Laboratory of Cell Pharmacology, University of Leuven Leuven, Belgium; University of Minnesota, United States of America

## Abstract

Enhanced Green Fluorescent Protein (EGFP) is the most commonly used live cell reporter despite a number of conflicting reports that it can affect cell physiology. Thus far, the precise mechanism of GFP-associated defects remained unclear. Here we demonstrate that EGFP and EGFP fusion proteins inhibit polyubiquitination, a posttranslational modification that controls a wide variety of cellular processes, like activation of kinase signalling or protein degradation by the proteasome. As a consequence, the NF-κB and JNK signalling pathways are less responsive to activation, and the stability of the p53 tumour suppressor is enhanced in cell lines and in vivo. In view of the emerging role of polyubiquitination in the regulation of numerous cellular processes, the use of EGFP as a live cell reporter should be carefully considered.

## Introduction

The extensive use of EGFP as a live cell reporter is based on the presumption that it does not affect (important) cellular functions. Nevertheless, a number of side effects have been reported. First a link between expression of GFP and induction of apoptosis was observed in number of cell lines [1]. In endothelial cells, the introduction of GFP by various gene transfer vectors selectively induced HSP70 resulting in the up-regulation of cyclooxygenase-2 (COX-2) expression followed by prostaglandin E2 production [2]. Recently, EGFP was shown to impair actin-myosin interactions in heart muscle cells [3], which might be linked to the dilated cardiomyopathy phenotype reported earlier on for GFP transgenic mice [4]. Furthermore, co-expression of EGFP and beta-galactosidase in neurons of transgenic mice induced neuropathology and premature death [5]. Up till now, the mechanism by which GFP provokes these defects remains undefined.

Ubiquitination is a posttranslational modification in which single or multiple ubiquitin molecules are attached to a protein. To date, two major forms of polyubiquitination have been functionally characterized, in which the isopeptide bond linkages involve Lys48 or Lys63. Typically, a polyubiquitin chain linked through Lys 48 targets a protein for degradation by the proteasome [6]. For example, the p53 tumour suppressor has to be maintained at a low steady-state level in most physiological conditions because of its inhibitory effect on cell growth. This duty is mainly fulfilled by MDM2, an E3 ubiquitin ligase that triggers constant degradation of p53 through Lys48-linked polyubiquitination [7], [8]. In contrast, Lys63-linked polyubiquitination serves a regulatory function via a degradation-independent mechanism and has been reported to control protein-kinase activation in NF-κB signalling [9].

In the present work we describe that EGFP blocks both Lys63- and Lys48-linked polyubiquitination and affects in this way NF-κB and JNK signalling and p53 homeostasis.

## Results and Discussion

**Figure 1:**
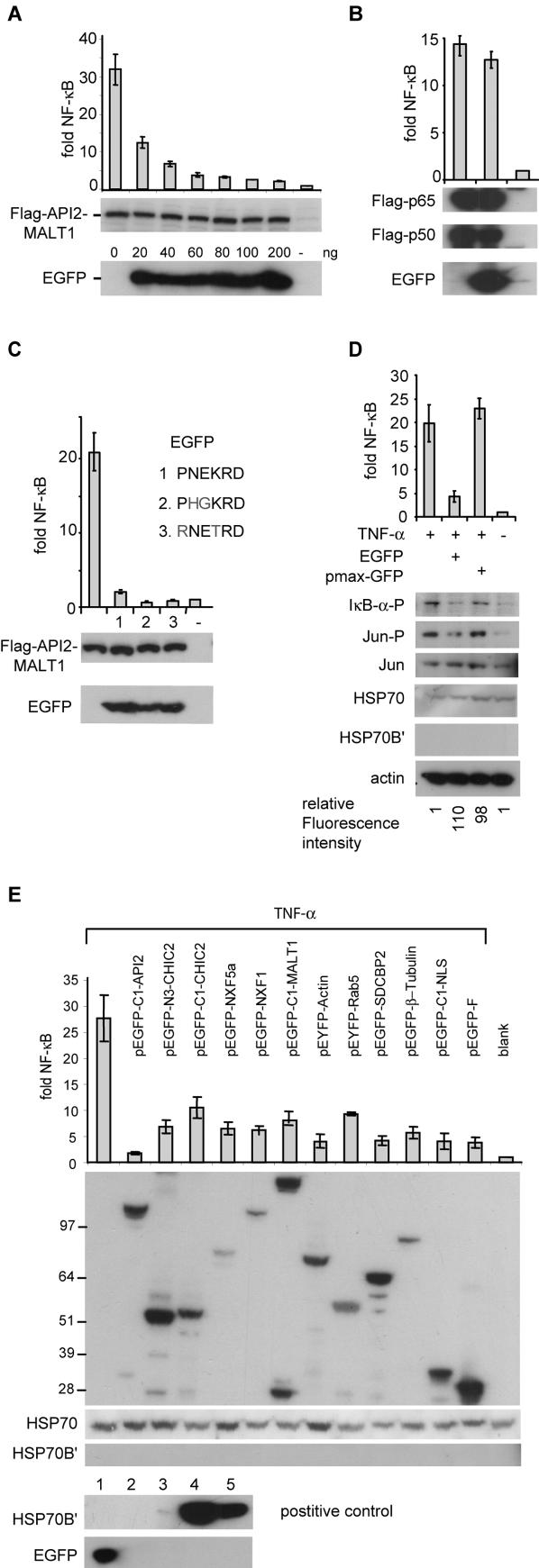
EGFP inhibits NF-κB and JNK activation in 293T cells (**A**) Activation of an NF-κB luciferase reporter by a Flag-tagged API2-MALT1 fusion protein is inhibited by EGFP in a dose dependent manner. (**B**) EGFP does not block NF-κB dependent luciferase activity induced by expression of the p50/p65 subunits of NF-κB (**C**) Activation of an NF-κB luciferase reporter by Flag-API2-MALT1 is inhibited by EGFP mutated for the TRAF6 binding motif (**D**) EGFP but not pmaxGFP prevents TNF-α induced activation of the NF-κB luciferase reporter and phosphorylation of IκB-α and JUN in 293T cells. EGFP does not increase HSP70 levels or induce HSP70B′ in 293T cells. Fluorescence intensities (Ex485/Em520nm) for EGFP and pmaxGFP were both ∼100 fold above background. (**E**) N- and/or C-terminal EGFP fusion proteins (for API2, CHIC2, NXF5a, NXF1, MALT1, Actin, Rab5, Syndecan binding protein 2 (SDCBP2) or β-Tubulin) and EGFP with a nuclear localization signal (NLS) or a farnysilation site (pEGFP-F) reduce TNF-α-induced NF-κB luciferase reporter activity in 293T cells. Bottom: In humans, HSP70 is constitutively expressed under normal conditions, but Hsp70B′ is only induced in response to stress. There is no basal expression of Hsp70B′[28]. As a positive controle for HSP70B′ expression, 293T cells (lane 2) were heat shocked at 44°C for two hours (lane 3) and allowed to recover at 37°C for 5 (lane 4) or 18 (lane 5) hours before harvest. NF-κB-dependent luciferase activity is represented for each experiment as fold induction of vector transfected cells and is represented graphically as the mean and standard deviation of at least three independent experiments. All molecular weight standards are in kDa.

Constitutive activation of the transcription factor NF-κB is considered essential for B-cell transformation by the API2-MALT1 fusion protein of MALT lymphomas with a translocation t(11;18) [10]. Transient overexpression of API2-MALT1 in 293T cells results in the robust activation of an NF-κB luciferase reporter gene [11], thereby providing a useful model system to study the mechanisms by which API2-MALT1 signals to NF-κB. To monitor for transfection efficiency, we co-expressed pEGFP-N2 (Clontech) in a number of experiments. To our surprise we observed that EGFP inhibited NF-κB activation induced by API2-MALT1 in a dose dependent manner (Fig 1A). In contrast, EGFP did not affect reporter activation induced by expression of the p50/p65 subunits of NF-κB (Fig 1B), suggesting an interference with the NF-κB signalling cascade activated by API2-MALT1 upstream of NF-κB.

MALT1 is an essential signalling component of the antigen-receptor pathway towards NF-κB [12], [13]. It is believed that BCL10 mediates oligomerization of MALT1, which allows the formation of a complex with TRAF6. Oligomerization of TRAF6 elicits the E3 ubiquitin ligase activity of its RING domain, resulting in modification of IKKγ (NEMO) via Lys63-linked polyubiquitin chains. This facilitates then the interaction of IKKγ with TGFβ activating kinase 1 (TAK1), which fully activates the IκB kinase complex (IKK) via phosphorylation of IKKβ [14], thereby leading to NF-κB activation. Similarly, the API2-MALT1 fusion protein activates NF-κB via TRAF6-mediated polyubiquitination of IKKγ [10], [15]. The interaction of TRAF6 with the Carboxy-terminus of MALT1 occurs via two potential TRAF6 binding motifs (PxExxAr/Ac with Ar/Ac for an aromatic or acidic residue [16]. Inspection of the EGFP sequence revealed the presence of a TRAF6 binding consensus (**P**N**E**KR**D**, AA 212–217). The crystal structure of EGFP shows that the PNEKRD motif is exposed in a loop between two beta-sheets [17], suggesting its accessibility and a role for EGFP as negative regulator via sequestering TRAF6. However, co-IP experiments failed to demonstrate an interaction between EGFP and TRAF6 (data not shown) and mutants for the TRAF6 binding consensus blocked NF-κB activation by API2-MALT1 as efficient as EGFP (Fig 1C).

To investigate whether the effect was limited to API2-MALT1, we analyzed TNF-α induced NF-κB activation in 293T cells expressing EGFP. Again, EGFP reduced NF-κB signalling as demonstrated by lower reporter activity and reduced phosphorylation of the inhibitor of NF-κB, IκB-α (Fig 1D). Next we evaluated whether EGFP fusion proteins might have the same effect. Both N- and C-terminal EGFP fusions blocked API2-MALT1- (data not shown) and TNFα-induced NF-κB activation (Fig 1E). Besides activating the NF-κB pathway, TNF-α treatment also triggers JNK signalling. Phosphorylation of JUN, indicative for activated JNK signalling, is reduced by EGFP as well after TNF-α treatment (Fig 1D).

It has been reported that prolonged visualization of GFP expressing cells induces the production of reactive oxygen species, which can result in physiological changes and eventually cell death [18]. Interestingly, both EGFP mutants for the TRAF6 binding consensus had lost their green fluorescence but efficiently inhibited NF-κB signalling (Fig 1C). Furthermore, pmaxGFP (Amaxa Biosystems), a structurally different fluorescent protein, did not effect NF-κB and JNK activation upon TNF-α treatment (Fig 1D), suggesting that the inhibition did not result from free-radical-associated phototoxicity. Another study indicated that high levels of EGFP can induce heat shock protein 70 (HSP70) [2], which is able to inhibit NF-κB activation via its interaction with IKKγ [19] and TRAF6 [20]. However, induction of HSP70 was restricted to endothelial cells [2] and we did not observe increased HSP70 levels or induction of HSP70B′ in EGFP expressing 293T cells (Fig 1D–E).

**Figure 2:**
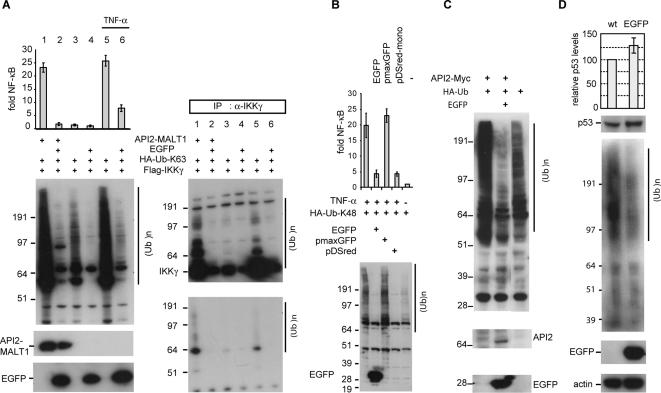
EGFP blocks Lys63- and Lys48-linked polyubiquitination. (**A**) 293T cells transfected with the indicated constructs and treated for 4 hours with 20 ng/ml TNF-α (lane 5 and 6) were immunoblotted with anti-Flag (API2-MALT1), anti-HA (HA-Ub-K63) and anti-EGFP antibodies (left panel) or anti-IKKγ immunoprecipitates were immunoblotted with anti-Flag (IKKγ) or anti-HA (ubiquitin) (right panel). (**B**) EGFP affects K48-linked polyubiquitination. 293T cells were transfected with a Ubiquitin construct with only Lys48 available for polymerization (HA-Ub-K48), treated for 4 hours with 20 ng/ml TNF-α or left untreated and cell lysates were immunoblotted with anti-HA (Ub-K48) and anti-EGFP antibodies. Fluorescence intensities (Excitation 485/Emission 520 nm) for EGFP and pmaxGFP were comparable (∼100 fold higher then background values), expression of pDs-Red was confirmed by Fluorescence microscopy. (**C**) EGFP stabilizes exogenous API2-Myc in 293T cells via reduction of its Lys48-linked auto-ubiquitination and proteasomal degradation. (**D**) Stable expression of EGFP in the merkel cell carcinoma cell line MCC14.2 reduces polyubiquitination and enhances endogenous p53 expression levels. The average ratio and standard deviation of p53 to actin signals are given (three independent experiments), (Ub)^n^ : polyubiquitinated proteins.

NF-κB activation by API2-MALT1 expression or upon TNF-α treatment is associated with modification of IKKγ with Lys63-linked polyubiquitin by TRAF6 or TRAF2 respectively [10], [21]. Furthermore TNFα-induced JNK signalling requires TRAF2 auto-ubiquitination [22]. To assess a possible effect of EGFP on the RING E3 ubiquitin ligase activity of TRAF6 or TRAF2, we monitored ubiquitination via a HA-tagged ubiquitin-mutant with only Lys63 available for polymerization (HA-Ub-K63). Expression of API2-MALT1 or TNF-α stimulation increased the level of polyubiquitinated proteins in 293T cells and was associated with enhanced IKKγ polyubiquitination in immunoprecipitates, and both processes were blocked by EGFP (Fig 2A, lanes 1,2,5,6). Moreover EGFP reduced the basal level of K63-linked polyubiquitination in 293T cells (Fig 2A, lanes 3 and 4). Similarly, expression of API2-MALT1 or TNFα treatment stimulates K48-linked ubiquitin chain assembly, which is again blocked by EGFP and its structural homologue dsRed (Clontech) but not by pmaxGFP (Fig 2B).

To investigate whether EGFP exclusively affects ubiquitination by TRAF proteins, we evaluated API2, a RING E3 ubiquitin ligase that destabilizes itself via Lys48-linked auto-ubiquitination [23]. Co-expression of EGFP inhibited polyubiquitination induced by API2 in 293T cells resulting in an increase of API2 expression levels (Fig 2C). Reduced steady-state levels of ubiquitination were also observed in MCC14.2 cells with stable expression of EGFP. As a consequence, basal levels of endogenous p53, which are tightly regulated through Lys48-linked ubiquitination by MDM2, are slightly increased in EGFP expressing cells (Fig 2D).

**Figure 3:**
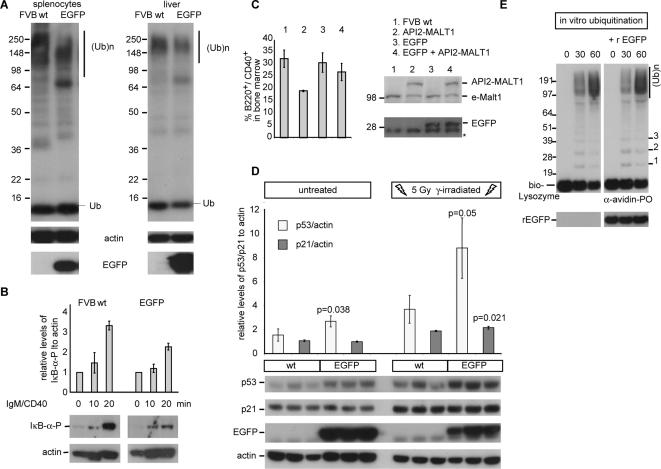
EGFP affects polyubiquitination-dependent processes ***in vivo***. (**A**) Western blots of spleen and liver extracts from FVB wild-type (wt) and EGFP transgenic mice detected with antibodies against Ubiquitin, EGFP and actin (loading control). (**B**) EGFP reduces phosphorylation of IκB-α in anti-IgM/anti-CD40 stimulated B-lymphocytes purified from EGFP mice. Experiments performed in triplicate, a representative image of one experiment is shown. The average and standard deviations of ratios of IκB-α-P to actin relative to un-stimulated cells are given. (**C**) The deficit of B220^+^/CD40^+^ B-cells in the bone marrow of API2-MALT1 mice is restored in EGFP/API2-MALT1 double transgenic mice. Experiments performed in triplicate, the average and standard deviations are depicted. eMalt1: endogenous Malt1, *a-specific band (**D**) EGFP mice show increased p53 levels in the liver and have enhanced p53/p21 responses upon γ-irradiation induced DNA damage. The average and standard deviations of the ratios of p53/p21 to actin signals relative to that of sample 1 are given. (**E**) Recombinant EGFP (rEGFP) does not prevent polyubiquitination of a Biotin-Lysozyme substrate *in vitro*. (Ub)^n^: polyubiquitinated proteins.

Next we examined whether EGFP affects ubiquitination *in vivo*. The basal levels of polyubiquitination were reduced in splenic lymphocytes from EGFP transgenic mice [24], which was associated with reduced phosphorylation of IκB-α following antigen stimulation compared to control mice, indicating that EGFP reduces B-cell receptor-induced NF-κB activation (Fig 3A–B). API2-MALT1 transgenic mice have a deficit of B220^+^/CD40^+^ B-cells in their bone marrow as API2-MALT1-mediated NF-κB activation accelerates their maturation to naïve B-cells [15]. When these API2-MALT1 transgenic mice were crossed with EGFP mice, the deficit of B220^+^/CD40^+^ B-cells in the bone marrow of EGFP/API2-MALT1 double transgenic mice (Fig 3C) was restored, further supporting an impact on ubiquitination and NF-κB signalling *in vivo*. Similarly, polyubiquitination was reduced in liver cells of EGFP mice and was associated with p53 stabilization as shown by the increase of its expression level (Fig 3A, D). The major target gene of p53 upon γ-irradiation-induced DNA damage in the liver is p21, which is required for cell cycle arrest and DNA repair [25]. Accordingly, γ-irradiation induced DNA damage caused not only a higher p53 response in EGFP mice but also the induction of p21 was slightly enhanced (Fig 3D). Finally *in vitro* experiments were performed to evaluate whether EGFP directly affects ubiquitin chain assembly; however recombinant EGFP did not affect polyubiquitination of a control substrate (Fig 3E), suggesting a cell context dependent effect of EGFP on ubiquitination.

In conclusion, our data indicate that EGFP and EGFP fusion proteins affect RING E3 ubiquitin ligase-dependent processes like NF-κB and JNK signalling or p53 homeostasis in cell lines and in mice. NF-κB plays a central role in the regulation of diverse biological processes, including innate and adaptive immunity, development, cell growth and survival [26]. The pro-survival signals result from the elevated expression of inhibitors of apoptosis, such as B-cell leukaemia/lymphoma-X_L._ Therefore, the increase in apoptosis induced by EGFP in cells [1] or in the motor cortex and striatum of the brain of mice [5] might be related to reduced NF-κB activity due to defective polyubiquitination and activation/degradation of its upstream regulators. Defective ubiquitination is also postulated to cause limb girdle muscular dystrophy, which is associated with mutations in Trim32, a ubiquitin ligase for actin [27]. Because EGFP was shown to impair actin-myosin interactions in muscle cells [3] and to induce dilated cardiomyopathy in EGFP mice [4], it is tempting to speculate that actin ubiquitination might play an essential role in normal muscle functioning and that its deregulation by EGFP causes the above mentioned phenotypes. Therefore, in view of the emerging role of ubiquitination in numerous cellular processes, the use of EGFP as a live cell reporter should be carefully considered.

## Materials and Methods

### Plasmids and reagents

The open reading frames of API2-MALT1, p50, p65 and IKKγ were amplified by RT-PCR using Long Distance PCR (Roche Applied Science) and cloned in pcDNA3.1 with an N-terminal Flag-tag. Ubiquitin and ubiquitin-mutants with only Lys48 or Lys63 available for polymerization were generated via PCR and cloned in pcDNA3.1 with an N-terminal HA-tag. pEGFP-N1 and pDsRed were from Clontech, pmaxGFP that encodes the green fluorescent protein (GFP) from Copepod Pontellina plumata from Amaxa Biosystems.

Antibodies specific for Ubiquitin (sc-8017), Actin (sc-8432), IKKγ (FL-419), p53 (DO-1) and HSP70 (K-20) were from Santa Cruz Biotechnology; anti-HSP70B′ from Stressgen, anti-EGFP and anti-Flag from Sigma-Aldrich, anti-HA (12CA5) from Roche Applied Science, and anti-p21 (DCS60), anti-Phospho-IκB-α (5A5), anti-Phospho-c-Jun (Ser63) (#9261) and anti-c-Jun (#9162) were from Cell Signaling.

### Cell culture, NF-κB reporter assays and IKKγ polyubiquitination

HEK293T were cultured in DMEM-F12 medium (Invitrogen) supplemented with 10% fetal calf serum at 37°C in 5% CO2. 5×10^5^ HEK293T-cells were transfected with 1 µg of DNA containing 20 ng of a NF-κB luciferase reporter (pIgκ3ConALuc), 20 ng of a β-galactosidase expression vector (pEL1-β-gal), a mixture of the expression constructs to test (2–200 ng) and the appropriate empty parental expression vector to keep the total amount of DNA at 1 µg per well of a six-well plate using Gene Juice transfection reagent (Novagen). 24 hours post-transfection, cells were harvested in 1× PBS, lysed in Passive lysis buffer (Promega) and assayed for luciferase and β-galactosidase activities using a Fluostar galaxy plate reader (BMG Labtechnologies). Fold NF-κB induction was calculated by dividing the luciferase activity, normalized to β-galactosidase activity for each sample, by that observed in the control containing only empty vector.

For the detection of IKKγ polyubiquitination, lysates from HEK293T-cells transfected with pcD-F-IKKγ/pcD-HA-Ub-K63 together with pcD-F-API2-MALT1 or empty vector or transfected with pcD-F-IKKγ/pcD-HA-Ub-K63 and treated for 4 hrs with 20 ng/ml TNFα (hBA-158, Santa Cruz) respectively were pre-cleared with protein G-sepharose (Amersham Biosciences) for 2 hours prior to immunoprecipitation overnight with an antibody against IKKγ. Immunoprecipitates were washed four times in lysis buffer, resolved by SDS-PAGE and detected with anti-HA or anti-Flag.

**Transgenic GFP mice** [24] had been backcrossed for at least 20 generations in strain FVB (Charles River laboratories) and bred as EGFP homozygotes. API2-MALT1 heterozygous transgenic mice generated in strain FVB [15] were crossed with EGFP transgenic mice to obtain EGFP/API2-MALT1 double transgenic and EGFP heterozygotes. All animal experiments were performed in accordance with institutional guidelines.

### IgM/CD40 stimulation of purified splenic B-lymphocytes

Single cell suspensions were prepared from spleens of healthy FVB and FVB-EGFP mice (12 weeks) and B-cells were isolated by negative selection (murine B-cell selection kit, Miltenyibiotech) according to the manufacturer's instructions. 15×10^6^ cells were stimulated with anti-IgM (10 µg/ml, Sigma-Aldrich) and anti-CD40 (2 µg/ml, BD Biosciences) for the indicated times.

### In vitro ubiquitination assay

The ubiquitination reactions were performed with the Ubiquitin-Protein Conjugation kit (K-960, Boston Biochemicals) with Biotin-Lysozyme as conjugation substrate (SP-100, Boston Biochemicals) in the presence of Ubiquitin aldehyde. Reactions were stopped by addition of reducing sample buffer and boiling for 1 min. Recombinant EGFP was purchased from Biovision.

### Flow cytometric analyses

Single-cell suspensions of bone marrow were labeled with PerCP-B220 and phycoerythrin-CD40 antibodies (BD Biosciences) and stained cells were analyzed with a FACSort (BD Biosciences) using the CELLQUEST software.
